# Supplementary material for: Genetic signatures of Mycobacterium tuberculosis Nonthaburi genotype revealed by whole genome analysis of isolates from tuberculous meningitis patients in Thailand
Source: PeerJ. 2016 Apr 12;4:e1905. doi: 10.7717/peerj.1905 (PMC4841212; doi:10.7717/peerj.1905)
Supplement: Figure S1 — SNPs common to isolates CSF-3053, 46-5069 and 43-13838 compared to 92,000 SNPs from 1,601 genomes of M. tuberculosis complex members (Coll et al., 2014a; Coll et al., 2014b) were used to position the isolates as belonging to subineage 1.2.1. [file peerj-04-1905-s001.pdf]

Supplementary Figure S1

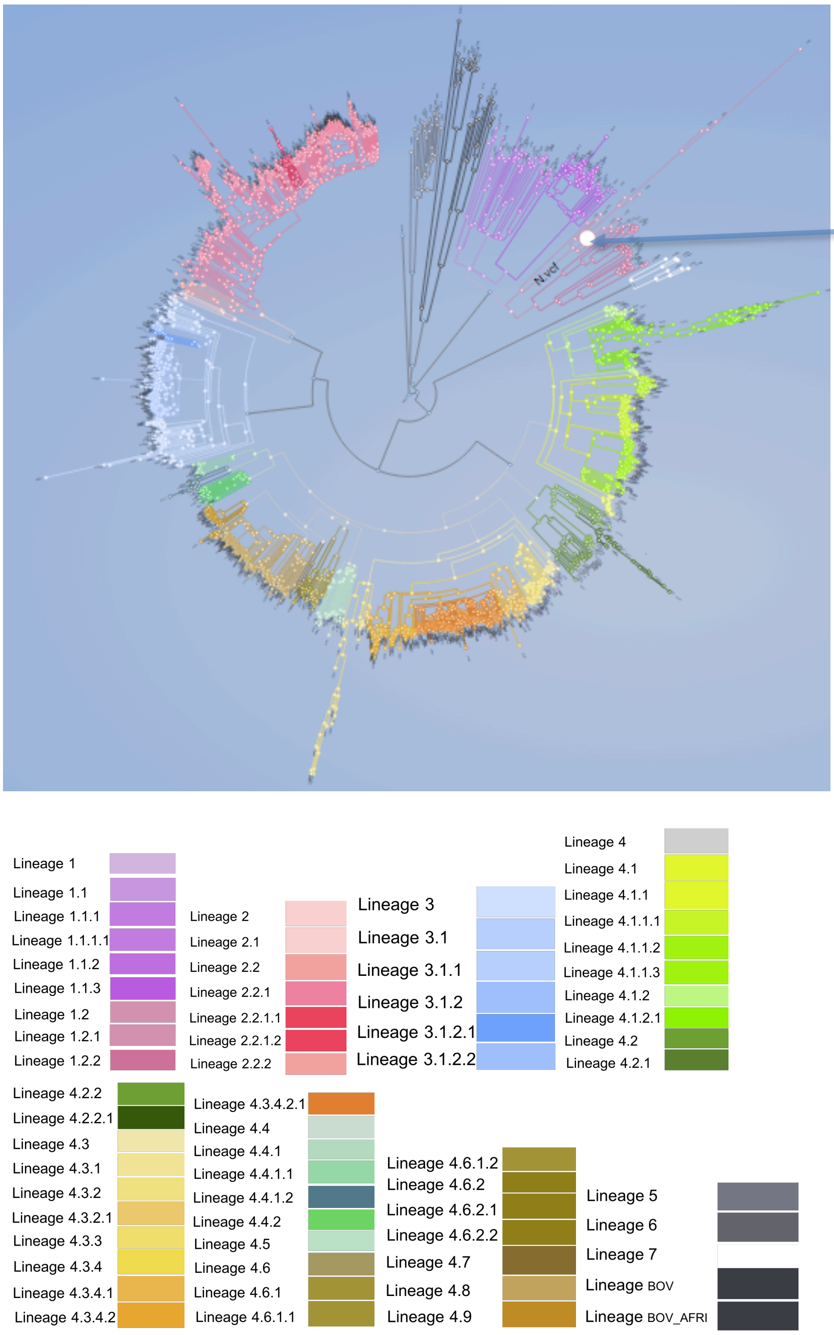

Position of isolates CSF-3053, 46-5069 and 43-13838 compared to 1,601 genome[illegible] of *M. tuberculosis* complex members.

Coll F, McNerney R, Guerra-Assuncao JA, Glynn JR, Perdigao J, Viveiros M, Portugal I, Pain A, Martin N, and Clark TG. 2014a. A robust SNP barcode for typing *Mycobacterium tuberculosis* complex strains. *Nature Communications* 5:4812. 10.1038/ncomms5812
