# Supplementary figures and images for: Genetic signatures of Mycobacterium tuberculosis Nonthaburi genotype revealed by whole genome analysis of isolates from tuberculous meningitis patients in Thailand
Source: PeerJ. 2016 Apr 12;4:e1905. doi: 10.7717/peerj.1905 (PMC4841212; doi:10.7717/peerj.1905)

**Figure S2**

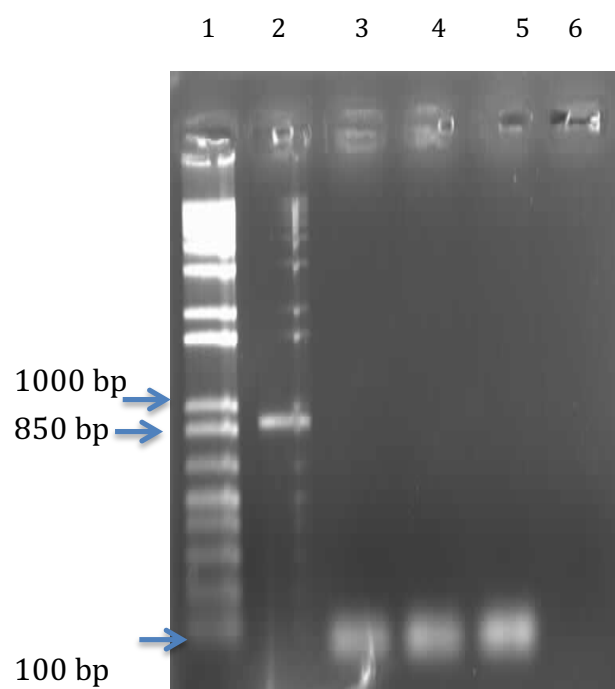

**Figure S3**

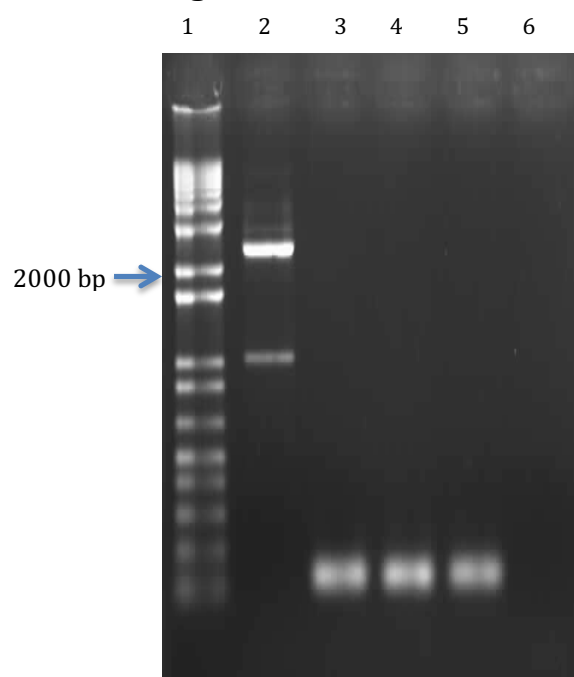

**Figure S4**

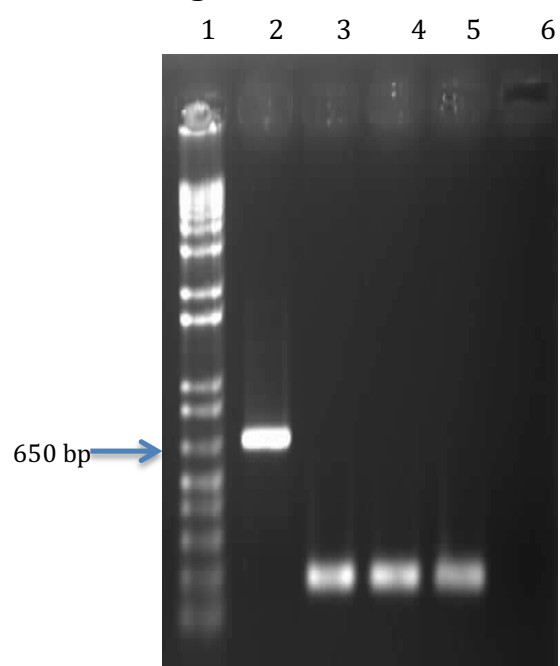

Supplement: Figures S2, S3 and S4 — Figure 2: PCR products using primers CF: GCCCAACCTGATTGGTTTCG and CR: CAAACGCTCGCCATGATCTC for RD239 Primers were designed to cover region 4092041-4092947. Expected size is 907 bp Lane 1: 1 kb DNA plus ladder Lane 2: M. tuberculosis (H37Rv, NC_000962.3) Lane 3: CSF3053 Lane 4: 46-5069 Lane 5: 43-13838. Figure 3: PCR products using primers AF: GCCCAACCTGATTGGTTTCG and AR: CAAACGCTCGCCATGATCTC for RD147c Primers were designed to cover region 1718833-1721268. Expected sixe is 2436 Lane 1: 1 kb DNA plus ladder Lane 2: M. tuberculosis (H37Rv NC_000962.3) Lane 3: CSF3053 Lane 4: 46-5069 Lane 5: 43-13838. Figure 4: PCR products using primers BF: GCCCAACCTGATTGGTTTCG and BR: CAAACGCTCGCCATGATCTC for 500 bp deletion. Primers were designed to cover region 3501124-3501822. Expected size is 699 bp Lane 1: 1 kb DNA plus ladder Lane 2: M. tuberculosis (H37Rv NC_000962.3) Lane 3: CSF3053 Lane 4: 46-5069 Lane 5: 43-13838. [file peerj-04-1905-s002.pdf]
