# Supplementary material for: Genetic signatures of Mycobacterium tuberculosis Nonthaburi genotype revealed by whole genome analysis of isolates from tuberculous meningitis patients in Thailand
Source: PeerJ. 2016 Apr 12;4:e1905. doi: 10.7717/peerj.1905 (PMC4841212; doi:10.7717/peerj.1905)
Supplement: Tables S1 and S2 — Table S1: The positions, nucleotide change, amino acid change and effect of single nucleotide polymorphisms in virulence genes that are common to isolates CSF3053, 46-5069 and 43-13838. The protein variation was determined by Protein Variation Effect Analyzer (PROVEAN), a web based protein variation analysis tool (Choi et al., 2012). Table S2: The positions, nucleotide change, amino acid change and effect of single nucleotide polymorphisms in efflux pump related genes that are common to isolates CSF3053, 46-5069 and 43-13838. The protein variation was determined by Protein Variation Effect Analyzer (PROVEAN), a web based protein variation analysis tool (Choi et al., 2012). [file peerj-04-1905-s003.docx]

**Single nucleotide polymorphisms in virulence genes and efflux pump related genes common to isolates CSF3053, 46-5069 and 43-13838**

**Table S1. Single nucleotide polymorphisms in virulence genes**

| Position | Nucleotide change | Amino acid change | Protein variation effect | Gene | Function/Description | | | Virulence References |  |  |
| --- | --- | --- | --- | --- | --- | --- | --- | --- | --- | --- |
| 200379 | C>T | Ala162Val | Deleterious | *mce1B* | Mammalian cell entry proteins | | | (Gioffre et al. 2005) |  |  |
| 206339 | T>C | Leu370Pro | Neutral | *mce1F* | Mammalian cell entry proteins | | | (Gioffre et al. 2005) |  |  |
| 206481 | C>G | Pro417Pro |  | *mce1F* | Mammalian cell entry proteins | | | (Gioffre et al. 2005; Pantel et al. 2012) |  |  |
| 206484 | G>T | Gly418Gly |  | *mce1F* | Mammalian cell entry proteins | | | (Gioffre et al. 2005) |  |  |
| 495198 | A>G | Phe706Ser | Deleterious | *pknG* | Protein kinase G | | | (Cowley et al. 2004) |  |  |
| 495473 | C>T | Ser614Ser |  | *pknG* | Protein kinase G | | | (Cowley et al. 2004) |  |  |
| 541201 | A>G | Leu97Leu |  | *mmpL4* | Membrane transport protein | | | (Domenech et al. 2005) |  |  |
| 580008 | A>G | Leu220Leu |  | *senX3* | Two component system sensor | | | (Rickman et al. 2004) |  |  |
| 580336 | C>A | Arg330Arg |  | *senX3* | Two component system sensor | | | (Rickman et al. 2004) |  |  |
| 686972 | T>C | Phe51Ser | Neutral | *mce2A* | Mammalian cell entry proteins | | | (Marjanovic et al. 2010) |  |  |
| 687602 | C>A | Thr261Asn | Deleterious | *mce2A* | Mammalian cell entry proteins | | | (Marjanovic et al. 2010) |  |  |
| 690248 | C>A | Thr397Asn | Neutral | *mce2C* | Mammalian cell entry proteins | | | (Marjanovic et al. 2010) |  |  |
| 690450 | A>C | Ala464Ala |  | *mce2C* | Mammalian cell entry proteins | | | (Marjanovic et al. 2010) |  |  |
| 690465 | T>G | Leu469Leu |  | *mce2C* | Mammalian cell entry proteins | | | (Marjanovic et al. 2010) |  |  |
| 1037012 | T>C | Met5Thr | Neutral | *pstA1* | Inorganic phosphate-ABC transporter | | | (Rengarajan et al. 2005) |  |  |
| 1037911 | C>T | Arg305* |  | *pstA1* | Inorganic phosphate-ABC transporter | | | (Rengarajan et al. 2005) |  |  |
| 1038500 | T>G | Gln472Pro | Neutral | *pknD* | Protein kinase D | | | (Be et al. 2012) |  |  |
| 1043136 | C>T | Thr341Ile | Deleterious | *pstS1* | Inorganic phosphate transporter | | | (Peirs et al. 2005) |  |  |
| 1079927 | C>A | Thr395Thr |  | *ctpV* | Copper efflux transporter P-type ATPase | | | (Ward et al. 2010) |  |  |
| 1080192 | G>A | Asp484Asn | Neutral | *ctpV* | Copper efflux transporter P-type ATPase | | | (Ward et al. 2010) |  |  |
| 1097023 | G>A | Gly70Ser | Deleterious | *mprA* | Two component system | | | (Zahrt & Deretic 2001) |  |  |
| 1098523 | T>A | Leu339His | Neutral | *mprB* | Two component system | | | (Zahrt & Deretic 2001) |  |  |
| 1443899 | T>C | Ala383Ala |  | *Rv1290c* | Conserved hypothetical protein | | | (McAdam et al. 2002) |  |  |
| 1694547 | A>C | Ter200Glu |  | *Rv1504c* | Conserved hypothetical protein | | | (Brodin et al. 2010) |  |  |
| 1726816 | G>T | Pro532Thr | Neutral | *pks5* | Probable Polyketide synthase | | | (Rousseau et al. 2003) |  |  |
| 1875886 | C>A | Leu195Met | Neutral | *pks7* | Probable Polyketide synthase | | | (Rousseau et al. 2003) | |  |
| 1876739 | T>G | Val479Gly | Deleterious | *pks7* | Probable Polyketide synthase | | | (Rousseau et al. 2003) | |  |
| 2066471 | T>C | Gly5Gly |  | *secA2* | Accessory SecA protein | | | (Kurtz et al. 2006) | |  |
| 2154724 | C>A | Arg463Leu | Neutral | *katG* | Catalase-peroxidase | | | (Ng et al. 2004; Wilson et al. 1995) | |  |
| 2209465 | G>A | Ala47Thr | Neutral | *Mce-3* | Mammalian cell entry proteins | | | (Senaratne et al. 2008) | |  |
| 2211826 | A>G | Lys67Lys |  | *mce3C* | Mammalian cell entry proteins | | | (Senaratne et al. 2008) | |  |
| 2215712 | G>A | Leu152Leu |  | *mce3F* | Mammalian cell entry proteins | | | (Senaratne et al. 2008) | |  |
| 2216370 | G>C | Gly372Arg | Deleterious | *mce3F* | Mammalian cell entry proteins | | | (Senaratne et al. 2008) | |  |
| 2216443 | C>A | Ala396Glu | Neutral | *mce3F* | Mammalian cell entry proteins | | | (Senaratne et al. 2008) | |  |
| 2222308 | T>C | Asp286Gly | Neutral | *Rv1979c* | Region of difference 2 | | | (Kozak et al. 2011) | |  |
| 2278507 | G>A | Ser142Ser |  | *hspX* | Dormancy associated protein | | | (Yuan et al. 1998) | |  |
| 2296042 | G>A | Pro3649Ala | Neutral | *pks12* | Probable Polyketide synthase | | | (Sirakova et al. 2003) | |  |
| 2631641 | T>G | Pro153Pro |  | *plcA* | Probable phospholipase C | | | (Raynaud et al. 2002) | |  |
| 2672514 | C>G | Leu1108Leu |  | *mbtB* | ABC iron transporter | | | (De Voss et al. 1999) | |  |
| 3244126 | | G>A | Val144Ile(N) | Neutral | *fadD26* | Fatty acid coA synthase | | | (Camacho et al. 1999; Cox et al. 1999; Rousseau et al. 2004) | |
| 3285318 | | C>T | Ala83Ala |  | *mmpl7* | Transmembrane transport protein | | | (Cox et al. 1999) | |
| 3293423 | | A>G | Asp977Asp |  | *pks1* | Probable Polyketide synthase | | | (Tsenova et al. 2005) | |
| 3296809 | | G>A | Asp344Asp |  | *pks15* | Probable Polyketide synthase | | | (Reed et al. 2004) | |
| 3447480 | | A>C | Leu316Arg(D) | Deleterious | *VirS* | Transcriptional regulator | | | (Singh et al. 2003) | |
| 3518167 | | A>G | Ile474Met(N) | Neutral | *nuoG* | Type I NADH dehydrogenase subunit | | | (Velmurugan et al. 2007) | |
| 3518555 | | A>G | Thr604Ala(N) | Neutral | *nuoG* | Type I NADH dehydrogenase subunit | | | (Velmurugan et al. 2007) | |
| 3984321 | | G>A | His375His |  | *cyp125* | Putative cytochrome P450 | | | (Chang et al. 2009) | |
| 3984926 | | G>A | Leu174Leu |  | *cyp125* | Putative cytochrome P450 | | | (Chang et al. 2009) | |
| 4112429 | | T>C | Ile363Val(N) | Neutral | *Rv3671c* | Serine protease | | | (Vandal et al. 2008) | |
| 4289953 | | A>G | Val526Ala(N)\ | Neutral | *mmpL8* | Integral membrane transport protein | | | (Converse et al. 2003) | |
| 4290564 | | G>T | Ala322Ala |  | *mmpL8* | Integral membrane transport protein | | | (Converse et al. 2003) | |
| 4290827 | | C>G | Gly235Arg(N) | Neutral | *mmpL8* | Integral membrane transport protein | | | (Converse et al. 2003) | |
| 4344058 | | T>C | Ser249Pro(D) | Deleterious | *Rv3868* | Secretion system Esx-1 C or S | | | (Lewis et al. 2003) | |
| 4345682 | | C>T | Ser215Leu(N) | Neutral | *Rv3869* | Secretion system Esx-1 C or S | | | (Lewis et al. 2003) | |
| 4348418 | | G>A | Leu646Leu |  | *Rv3870* | Secretion system Esx-1 C or S | | | (Lewis et al. 2003) | |

The positions, nucleotide change , amino acid change and effect of single nucleotide polymorphisms in virulence genes that are common to isolates CSF3053, 46-5069 and 43-13838. The protein variation was determined by Protein Variation Effect Analyzer (PROVEAN), a web based protein variation analysis tool (Choi et al. 2012)

**Table S2 Single nucleotide polymorphisms in efflux pump related genes**

| Position | Nucleotide change | Amino acid change | Protein variation effect | Gene | Reference |
| --- | --- | --- | --- | --- | --- |
| 41151 | C>A | Ala18Ser | Neutral | *Rv0037c* | (De Rossi et al. 2002) |
| 227098 | T>C | Met74Thr | Neutral | *Rv0194* | (Danilchanka et al. 2008) |
| 412280 | T>G | His481Gln | Neutral | *iniA* | (Colangeli et al. 2005) |
| 541201 | A>G | Leu97Leu |  | *mmpL4* | (Domenech et al. 2005) |
| 938246 | A>G | Leu45Leu |  | *Rv0842* | (Li et al. 2015) |
| 947176 | C>G | Ala374Gly | Neutral | *Rv0849* | (De Rossi et al. 2002) |
| 1362006 | T>C | Gln243Arg | Neutral | *Rv1218c* | (Balganesh et al. 2010) |
| 1395010 | A>G | Arg278Gly | Neutral | *Rv1250* | (De Rossi et al. 2002) |
| 1406312 | A>G | His343His |  | *Rv1258c* | (Ainsa et al. 1998) |
| 1839260 | T>G | Leu31Leu |  | *Rv1634* | (De Rossi et al. 2002) |
| 1839329 | G>A | Arg54Arg |  | *Rv1634* | (De Rossi et al. 2002) |
| 1839759 | G>C | Gly198Arg | Neutral | *Rv1634* | (De Rossi et al. 2002) |
| 1897646 | G>C | Pro188Ala | Deleterious | *Rv1672c* | (Li et al. 2015) |
| 1897938 | G>A | Val90Val |  | *Rv1672c* | (Li et al. 2015) |
| 2062922 | T>C | Ile603Val | Neutral | *Rv1819c* | (Gupta et al. 2006) |
| 2127523 | G>A | Leu540Leu |  | *Rv1877* | (De Rossi et al. 2002) |
| 2538793 | G>A | Gly32Ser | Neutral | *Rv2265* | (Zhang et al. 2013) |
| 2608117 | G>T | Asp69Tyr | Neutral | *Rv2333c* | (Ramon-Garcia et al. 2007) |
| 3005185 | G>T | Pro156Thr | Neutral | *Rv2688c* | (Pasca et al. 2004) |
| 3154414 | A>G | Ile73Thr | Neutral | *efpA* | (Doran et al. 1997) |
| 3273138 | C>G | His309Asp | Neutral | *drrA* | (Choudhuri et al. 2002) |
| 3274545 | G>A | Leu158Leu |  | *drrC* | (Choudhuri et al. 2002) |
| 3285318 | C>T | Ala83Ala |  | *mmpL7* | (Pasca et al. 2005) |
| 3351472 | G>A | Trp68* |  | *Rv2994* | (De Rossi et al. 2002) |
| 3614982 | T>C | Leu874Leu |  | *Rv3239c* | (De Rossi et al. 2002) |

The positions, nucleotide change , amino acid change and effect of single nucleotide polymorphisms in efflux pump related genes that are common to isolates CSF3053, 46-5069 and 43-13838. The protein variation was determined by Protein Variation Effect Analyzer (PROVEAN), a web based protein variation analysis tool (Choi et al. 2012)

**References**

Ainsa JA, Blokpoel MC, Otal I, Young DB, De Smet KA, and Martin C. 1998. Molecular cloning and characterization of Tap, a putative multidrug efflux pump present in *Mycobacterium fortuitum* and *Mycobacterium tuberculosis*. *Journal of Bacteriology* 180:5836-5843.

Balganesh M, Kuruppath S, Marcel N, Sharma S, Nair A, and Sharma U. 2010. Rv1218c, an ABC transporter of Mycobacterium tuberculosis with implications in drug discovery. *Antimicrobial agents and chemotherapy* 54:5167-5172. 10.1128/AAC.00610-10

Be NA, Bishai WR, and Jain SK. 2012. Role of *Mycobacterium tuberculosis* pknD in the pathogenesis of central nervous system tuberculosis. *BMC microbiology* 12:7. 10.1186/1471-2180-12-7

Brennan MJ, Delogu G, Chen Y, Bardarov S, Kriakov J, Alavi M, and Jacobs WR, Jr. 2001. Evidence that mycobacterial PE_PGRS proteins are cell surface constituents that influence interactions with other cells. *Infection and Immunity* 69:7326-7333. 10.1128/IAI.69.12.7326-7333.2001

Brodin P, Poquet Y, Levillain F, Peguillet I, Larrouy-Maumus G, Gilleron M, Ewann F, Christophe T, Fenistein D, Jang J, Jang MS, Park SJ, Rauzier J, Carralot JP, Shrimpton R, Genovesio A, Gonzalo-Asensio JA, Puzo G, Martin C, Brosch R, Stewart GR, Gicquel B, and Neyrolles O. 2010. High content phenotypic cell-based visual screen identifies *Mycobacterium tuberculosis* acyltrehalose-containing glycolipids involved in phagosome remodeling. *PLoS Pathogens* 6:e1001100. 10.1371/journal.ppat.1001100

Camacho LR, Ensergueix D, Perez E, Gicquel B, and Guilhot C. 1999. Identification of a virulence gene cluster of *Mycobacterium tuberculosis* by signature-tagged transposon mutagenesis. *Molecular Microbiology* 34:257-267.

Chang JC, Miner MD, Pandey AK, Gill WP, Harik NS, Sassetti CM, and Sherman DR. 2009. igr Genes and *Mycobacterium tuberculosis* cholesterol metabolism. *Journal of Bacteriology* 191:5232-5239. 10.1128/JB.00452-09

1. Choi Y, Sims GE, Murphy S, Miller JR and Chan AP. 2012. [Predicting the Functional Effect of Amino Acid Substitutions and Indels](http://dx.plos.org/10.1371/journal.pone.0046688). *PLoS ONE* 7(10): e46688.

Choudhuri BS, Bhakta S, Barik R, Basu J, Kundu M, and Chakrabarti P. 2002. Overexpression and functional characterization of an ABC (ATP-binding cassette) transporter encoded by the genes drrA and drrB of *Mycobacterium tuberculosis. The Biochemical Journal* 367:279-285. 10.1042/BJ20020615

Colangeli R, Helb D, Sridharan S, Sun J, Varma-Basil M, Hazbon MH, Harbacheuski R, Megjugorac NJ, Jacobs WR, Jr., Holzenburg A, Sacchettini JC, and Alland D. 2005. The *Mycobacterium tuberculosis* iniA gene is essential for activity of an efflux pump that confers drug tolerance to both isoniazid and ethambutol. *Molecular Microbiology* 55:1829-1840. 10.1111/j.1365-2958.2005.04510.x

Converse SE, Mougous JD, Leavell MD, Leary JA, Bertozzi CR, and Cox JS. 2003. MmpL8 is required for sulfolipid-1 biosynthesis and *Mycobacterium tuberculosis* virulence. *Proceedings of the National Academy of Sciences of the United States of America* 100:6121-6126. 10.1073/pnas.1030024100

Cowley S, Ko M, Pick N, Chow R, Downing KJ, Gordhan BG, Betts JC, Mizrahi V, Smith DA, Stokes RW, and Av-Gay Y. 2004. The *Mycobacterium tuberculosis* protein serine/threonine kinase PknG is linked to cellular glutamate/glutamine levels and is important for growth in vivo. *Molecular Microbiology* 52:1691-1702. 10.1111/j.1365-2958.2004.04085.x

Cox JS, Chen B, McNeil M, and Jacobs WR, Jr. 1999. Complex lipid determines tissue-specific replication of *Mycobacterium tuberculosis* in mice. *Nature* 402:79-83. 10.1038/47042

Danilchanka O, Mailaender C, and Niederweis M. 2008. Identification of a novel multidrug efflux pump of *Mycobacterium tuberculosis*. *Antimicrobial Agents and Chemotherapy* 52:2503-2511. 10.1128/AAC.00298-08

De Rossi E, Arrigo P, Bellinzoni M, Silva PA, Martin C, Ainsa JA, Guglierame P, and Riccardi G. 2002. The multidrug transporters belonging to major facilitator superfamily in *Mycobacterium tuberculosis*. *Molecular Medicine* 8:714-724.

De Voss JJ, Rutter K, Schroeder BG, and Barry CE, 3rd. 1999. Iron acquisition and metabolism by mycobacteria. *Journal of Bacteriology* 181:4443-4451.

Domenech P, Reed MB, and Barry CE, 3rd. 2005. Contribution of the *Mycobacterium tuberculosis* MmpL protein family to virulence and drug resistance. *Infection and Immunity* 73:3492-3501. 10.1128/IAI.73.6.3492-3501.2005

Doran JL, Pang Y, Mdluli KE, Moran AJ, Victor TC, Stokes RW, Mahenthiralingam E, Kreiswirth BN, Butt JL, Baron GS, Treit JD, Kerr VJ, Van Helden PD, Roberts MC, and Nano FE. 1997. *Mycobacterium tuberculosis* *efpA* encodes an efflux protein of the QacA transporter family. *Clinical and Diagnostic Laboratory Immunology* 4:23-32.

Gioffre A, Infante E, Aguilar D, Santangelo MP, Klepp L, Amadio A, Meikle V, Etchechoury I, Romano MI, Cataldi A, Hernandez RP, and Bigi F. 2005. Mutation in mce operons attenuates *Mycobacterium tuberculosis* virulence. *Microbes and Infection / Institut Pasteur* 7:325-334. 10.1016/j.micinf.2004.11.007

Gupta AK, Chauhan DS, Srivastava K, Das R, Batra S, Mittal M, Goswami P, Singhal N, Sharma VD, Venkatesan K, Hasnain SE, and Katoch VM. 2006. Estimation of efflux mediated multi-drug resistance and its correlation with expression levels of two major efflux pumps in mycobacteria. *The Journal of Communicable Diseases* 38:246-254.

Kozak RA, Alexander DC, Liao R, Sherman DR, and Behr MA. 2011. Region of difference 2 contributes to virulence of *Mycobacterium tuberculosis. Infection and Immunity* 79:59-66. 10.1128/IAI.00824-10

Kurtz S, McKinnon KP, Runge MS, Ting JP, and Braunstein M. 2006. The SecA2 secretion factor of *Mycobacterium tuberculosis* promotes growth in macrophages and inhibits the host immune response. *Infection and Immunity* 74:6855-6864. 10.1128/IAI.01022-06

Lewis KN, Liao R, Guinn KM, Hickey MJ, Smith S, Behr MA, and Sherman DR. 2003. Deletion of RD1 from *Mycobacterium tuberculosis* mimics bacille Calmette-Guerin attenuation. *The Journal of Infectious Diseases* 187:117-123. 10.1086/345862

Li G, Zhang J, Guo Q, Wei J, Jiang Y, Zhao X, Zhao LL, Liu Z, Lu J, and Wan K. 2015. Study of efflux pump gene expression in rifampicin-monoresistant *Mycobacterium tuberculosis* clinical isolates. *The Journal of Antibiotics*. 10.1038/ja.2015.9

Marjanovic O, Miyata T, Goodridge A, Kendall LV, and Riley LW. 2010. Mce2 operon mutant strain of *Mycobacterium tuberculosis* is attenuated in C57BL/6 mice. *Tuberculosis* 90:50-56. 10.1016/j.tube.2009.10.004

Master SS, Springer B, Sander P, Boettger EC, Deretic V, and Timmins GS. 2002. Oxidative stress response genes in *Mycobacterium tuberculosis*: role of ahpC in resistance to peroxynitrite and stage-specific survival in macrophages. *Microbiology* 148:3139-3144.

McAdam RA, Quan S, Smith DA, Bardarov S, Betts JC, Cook FC, Hooker EU, Lewis AP, Woollard P, Everett MJ, Lukey PT, Bancroft GJ, Jacobs Jr WR, Jr., and Duncan K. 2002. Characterization of a *Mycobacterium tuberculosis* H37Rv transposon library reveals insertions in 351 ORFs and mutants with altered virulence. *Microbiology* 148:2975-2986.

Ng VH, Cox JS, Sousa AO, MacMicking JD, and McKinney JD. 2004. Role of KatG catalase-peroxidase in mycobacterial pathogenesis: countering the phagocyte oxidative burst. *Molecular Microbiology* 52:1291-1302. 10.1111/j.1365-2958.2004.04078.x

Pantel A, Petrella S, Veziris N, Brossier F, Bastian S, Jarlier V, Mayer C, and Aubry A. 2012. Extending the definition of the GyrB quinolone resistance-determining region in *Mycobacterium tuberculosi*s DNA gyrase for assessing fluoroquinolone resistance in M. tuberculosis. *Antimicrobial Agents and Chemotherapy* 56:1990-1996. 10.1128/AAC.06272-11

Pasca MR, Guglierame P, Arcesi F, Bellinzoni M, De Rossi E, and Riccardi G. 2004. Rv2686c-Rv2687c-Rv2688c, an ABC fluoroquinolone efflux pump in *Mycobacterium tuberculosis*. *Antimicrobial agents and chemotherapy* 48:3175-3178. 10.1128/AAC.48.8.3175-3178.2004

Pasca MR, Guglierame P, De Rossi E, Zara F, and Riccardi G. 2005. mmpL7 gene of Mycobacterium tuberculosis is responsible for isoniazid efflux in *Mycobacterium smegmatis. Antimicrobial Agents and Chemotherapy* 49:4775-4777. 10.1128/AAC.49.11.4775-4777.2005

Peirs P, Lefevre P, Boarbi S, Wang XM, Denis O, Braibant M, Pethe K, Locht C, Huygen K, and Content J. 2005. *Mycobacterium tuberculosis* with disruption in genes encoding the phosphate binding proteins PstS1 and PstS2 is deficient in phosphate uptake and demonstrates reduced in vivo virulence. *Infection and Immunity* 73:1898-1902. 10.1128/IAI.73.3.1898-1902.2005

Ramon-Garcia S, Martin C, De Rossi E, and Ainsa JA. 2007. Contribution of the Rv2333c efflux pump (the Stp protein) from *Mycobacterium tuberculosis* to intrinsic antibiotic resistance in *Mycobacterium bovis* BCG. *The Journal of Antimicrobial Chemotherapy* 59:544-547. 10.1093/jac/dkl510

Raynaud C, Guilhot C, Rauzier J, Bordat Y, Pelicic V, Manganelli R, Smith I, Gicquel B, and Jackson M. 2002. Phospholipases C are involved in the virulence of *Mycobacterium tuberculosis*. *Molecular Microbiology* 45:203-217.

Reed MB, Domenech P, Manca C, Su H, Barczak AK, Kreiswirth BN, Kaplan G, and Barry CE, 3rd. 2004. A glycolipid of hypervirulent tuberculosis strains that inhibits the innate immune response. *Nature* 431:84-87. 10.1038/nature02837

Rengarajan J, Bloom BR, and Rubin EJ. 2005. Genome-wide requirements for *Mycobacterium tuberculosis* adaptation and survival in macrophages. *Proceedings of the National Academy of Sciences of the United States of America* 102:8327-8332. 10.1073/pnas.0503272102

Rickman L, Saldanha JW, Hunt DM, Hoar DN, Colston MJ, Millar JB, and Buxton RS. 2004. A two-component signal transduction system with a PAS domain-containing sensor is required for virulence of *Mycobacterium tuberculosis* in mice. *Biochemical and Biophysical Research Communications* 314:259-267.

Rousseau C, Sirakova TD, Dubey VS, Bordat Y, Kolattukudy PE, Gicquel B, and Jackson M. 2003. Virulence attenuation of two Mas-like polyketide synthase mutants of *Mycobacterium tuberculosis*. *Microbiology* 149:1837-1847.

Rousseau C, Winter N, Pivert E, Bordat Y, Neyrolles O, Ave P, Huerre M, Gicquel B, and Jackson M. 2004. Production of phthiocerol dimycocerosates protects *Mycobacterium tuberculosis* from the cidal activity of reactive nitrogen intermediates produced by macrophages and modulates the early immune response to infection. *Cellular Microbiology* 6:277-287.

Senaratne RH, Sidders B, Sequeira P, Saunders G, Dunphy K, Marjanovic O, Reader JR, Lima P, Chan S, Kendall S, McFadden J, and Riley LW. 2008. *Mycobacterium tuberculosis* strains disrupted in mce3 and mce4 operons are attenuated in mice. *Journal of Medical Microbiology* 57:164-170. 10.1099/jmm.0.47454-0

Singh A, Jain S, Gupta S, Das T, and Tyagi AK. 2003. mymA operon of *Mycobacterium tuberculosis*: its regulation and importance in the cell envelope. *FEMS Microbiology letters* 227:53-63.

Sirakova TD, Dubey VS, Kim HJ, Cynamon MH, and Kolattukudy PE. 2003. The largest open reading frame (pks12) in the *Mycobacterium* *tuberculosis* genome is involved in pathogenesis and dimycocerosyl phthiocerol synthesis. *Infection and Immunity* 71:3794-3801.

Tsenova L, Ellison E, Harbacheuski R, Moreira AL, Kurepina N, Reed MB, Mathema B, Barry CE, 3rd, and Kaplan G. 2005. Virulence of selected *Mycobacterium tuberculosis* clinical isolates in the rabbit model of meningitis is dependent on phenolic glycolipid produced by the bacilli. *The Journal of Infectious Diseases* 192:98-106. 10.1086/430614

Vandal OH, Pierini LM, Schnappinger D, Nathan CF, and Ehrt S. 2008. A membrane protein preserves intrabacterial pH in intraphagosomal *Mycobacterium tuberculosis*. *Nature Medicine* 14:849-854. 10.1038/nm.1795

Velmurugan K, Chen B, Miller JL, Azogue S, Gurses S, Hsu T, Glickman M, Jacobs WR, Jr., Porcelli SA, and Briken V. 2007. *Mycobacterium* *tuberculosis nuoG* is a virulence gene that inhibits apoptosis of infected host cells. *PLoS Pathogens* 3:e110. 10.1371/journal.ppat.0030110

Ward SK, Abomoelak B, Hoye EA, Steinberg H, and Talaat AM. 2010. CtpV: a putative copper exporter required for full virulence of *Mycobacterium tuberculosis. Molecular Microbiology* 77:1096-1110. 10.1111/j.1365-2958.2010.07273.x

Wilson T, de Lisle GW, Marcinkeviciene JA, Blanchard JS, and Collins DM. 1998. Antisense RNA to ahpC, an oxidative stress defence gene involved in isoniazid resistance, indicates that AhpC of *Mycobacterium bovis* has virulence properties. *Microbiology* 144 ( Pt 10):2687-2695.

Wilson TM, de Lisle GW, and Collins DM. 1995. Effect of inhA and katG on isoniazid resistance and virulence of *Mycobacterium bovis*. *Molecular Microbiology* 15:1009-1015.

Yuan Y, Crane DD, Simpson RM, Zhu YQ, Hickey MJ, Sherman DR, and Barry CE, 3rd. 1998. The 16-kDa alpha-crystallin (Acr) protein of *Mycobacterium tuberculosis* is required for growth in macrophages. *Proceedings of the National Academy of Sciences of the United States of America* 95:9578-9583.

Zahrt TC, and Deretic V. 2001. *Mycobacterium tuberculosis* signal transduction system required for persistent infections. *Proceedings of the National Academy of Sciences of the United States of America* 98:12706-12711. 10.1073/pnas.221272198

Zhang JR, Li GL, Zhao XQ, Wan KL, and Lu JX. 2013. [A primary investigation on the isoniazid-induced alterations in efflux gene expression among the isoniazid resistant *Mycobacterium tuberculosis* clinical isolates]. *Zhonghua liu xing bing xue za zhi = Zhonghua liuxingbingxue zazhi* 34:379-384.
